# (*E*)-3,3,6,6-Tetra­methyl-9-(2-nitro­styr­yl)-3,4,5,6,7,9-hexa­hydro-1*H*-xanthene-1,8(2*H*)-dione

**DOI:** 10.1107/S1600536812023495

**Published:** 2012-05-31

**Authors:** Jae Kyun Lee, Sun-Joon Min, Yong Seo Cho, Ki Soo Lee, Joo Hwan Cha

**Affiliations:** a Center for Neuro-Medicine, Korea Institute of Science and Technology, Hwarangro 14-gil, Seongbuk-gu, Seoul 136-791, Republic of Korea; b Advanced Analysis Center, Korea Institute of Science and Technology, Hwarangro 14-gil, Seongbuk-gu, Seoul 136-791, Republic of Korea

## Abstract

In the title compound, C_25_H_27_NO_5_, each of the cyclo­hexenone rings adopts a half-chair conformation, whereas the six-membered pyran ring adopts a flattened boat conformation, with the O and methine C atoms deviating from the plane of the other four atoms. In the crystal, weak C—H⋯O hydrogen bonds link mol­ecules into chains parallel to the *c* axis.

## Related literature

For the crystal structures of xanthene derivatives studied recently by our group, see: Cha *et al.* (2012 ▶); Lee *et al.* (2012 ▶).
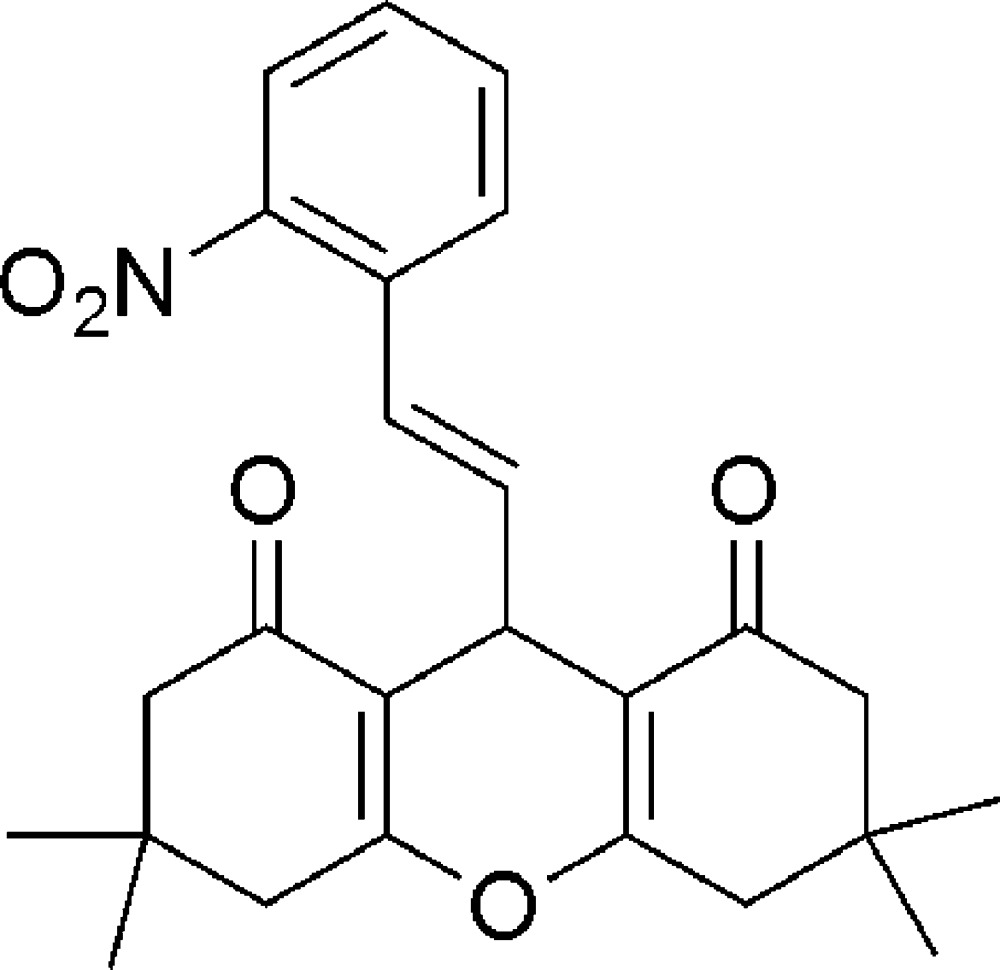


## Experimental

### 

#### Crystal data

C_25_H_27_NO_5_
*M* *_r_* = 421.49
Monoclinic, 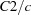

*a* = 33.312 (3) Å
*b* = 9.4144 (6) Å
*c* = 14.4581 (10) Å
β = 102.3931 (19)°
*V* = 4428.6 (6) Å^3^
*Z* = 8
Mo *K*α radiation
μ = 0.09 mm^−1^
*T* = 296 K
0.30 × 0.30 × 0.30 mm

#### Data collection

Rigaku R-AXIS RAPID diffractometer
Absorption correction: multi-scan (*ABSCOR*; Higashi, 1995 ▶) *T*_min_ = 0.753, *T*_max_ = 0.974
20666 measured reflections
5066 independent reflections
3965 reflections with *F* ^2^ > 2.0σ(*F* ^2^)
*R*_int_ = 0.022

#### Refinement

*R*[*F* ^2^ > 2σ(*F* ^2^)] = 0.048
*wR*(*F* ^2^) = 0.153
*S* = 1.05
5066 reflections
288 parameters
H atoms treated by a mixture of independent and constrained refinement
Δρ_max_ = 0.32 e Å^−3^
Δρ_min_ = −0.27 e Å^−3^

### 

Data collection: *RAPID-AUTO* (Rigaku, 2006 ▶); cell refinement: *RAPID-AUTO*; data reduction: *RAPID-AUTO*; program(s) used to solve structure: *SIR2008* in *Il Milione* (Burla *et al.*, 2007 ▶); program(s) used to refine structure: *SHELXL97* (Sheldrick, 2008 ▶); molecular graphics: *CrystalStructure* (Rigaku, 2010 ▶); software used to prepare material for publication: *CrystalStructure*.

## Supplementary Material

Crystal structure: contains datablock(s) global, I. DOI: 10.1107/S1600536812023495/ff2067sup1.cif

Structure factors: contains datablock(s) I. DOI: 10.1107/S1600536812023495/ff2067Isup2.hkl

Supplementary material file. DOI: 10.1107/S1600536812023495/ff2067Isup3.cml

Additional supplementary materials:  crystallographic information; 3D view; checkCIF report

## Figures and Tables

**Table 1:** Hydrogen-bond geometry (Å, °)

*D*—H⋯*A*	*D*—H	H⋯*A*	*D*⋯*A*	*D*—H⋯*A*
C17—H17⋯O5^i^	0.93	2.49	3.420 (3)	176

Symmetry code: (i) 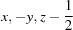.

**Fig. 1.**
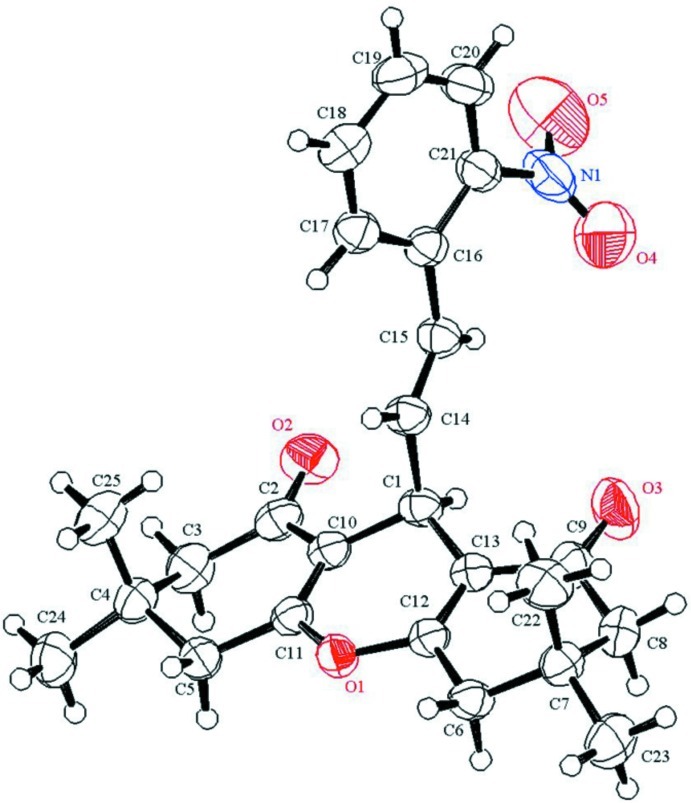
The molecular structure of (I) showing the atomic numbering and 50% probability displacement ellipsoid.

## supplementary crystallographic information

### Comment

As part of our ongoing study of the substituent effect on the solid state structures of xanthene derivatives (Cha *et al.*, (2012). We present here the crystal structure of the title compound (I) (Fig. 1).

In (I) (Fig. 1), the bond lengths and angles are normal and correspond to those observed in related structures (Cha *et al.*, 2012). All two cyclohexenone rings in (I) display half-chair conformation, whereas the pyran ring adopts a boat conformation.

In the crystal, weak intermolecular C17—H17···O5 hydrogen bonds into chains parallel to the *c* axis.

### Experimental

To solution of (*E*)-2.2-(3-(2-Nitrophenyl)prop-2-ene-1,1-diyl)bis (3-hydroxy-5,5-δimethylcyclohex-2-enone) (1.25 mmol) in methanol (12.5 ml) was added catalytic amount of sulfuric acid under nitrogen atmosphere. After stirring for 3 h, the solvent was evaporated and the remaining residue dissolved in ethyl acetate. The mixture was neutralized with saturated sodium bicarbonate and the solution was extracted with ethyl acetate. The resulting residue solid was purified by recrystallization from a mixture of ethanol and methylene chloride (1:1) to afford colorless block type crystals suitable for X-ray analysis.

### Refinement

All hydrogen atoms were positioned geometrically and refined using a riding model with C—H = 0.93–1.00 Å and *U*_iso_(H) = 1.2 or 1.5 *U*eq(C).

### Crystal data

**Table:**

C_25_H_27_NO_5_	*F*(000) = 1792.00
*M**_r_* = 421.49	*D*_x_ = 1.264 Mg m^−^^3^
Monoclinic, *C*2/*c*	Mo *K*α radiation, λ = 0.71075 Å
Hall symbol: -C 2yc	Cell parameters from 15305 reflections
*a* = 33.312 (3) Å	θ = 3.0–27.5°
*b* = 9.4144 (6) Å	µ = 0.09 mm^−^^1^
*c* = 14.4581 (10) Å	*T* = 296 K
β = 102.3931 (19)°	Block, colourless
*V* = 4428.6 (6) Å^3^	0.30 × 0.30 × 0.30 mm
*Z* = 8	

### Data collection

**Table:**

Rigaku R-AXIS RAPID diffractometer	3965 reflections with *F*^2^ > 2.0σ(*F*^2^)
Detector resolution: 10.000 pixels mm^-1^	*R*_int_ = 0.022
ω scans	θ_max_ = 27.5°
Absorption correction: multi-scan (*ABSCOR*; Rigaku, 1995)	*h* = −43→43
*T*_min_ = 0.753, *T*_max_ = 0.974	*k* = −11→12
20666 measured reflections	*l* = −18→15
5066 independent reflections	

### Refinement

**Table:**

Refinement on *F*^2^	Secondary atom site location: difference Fourier map
*R*[*F*^2^ > 2σ(*F*^2^)] = 0.048	Hydrogen site location: inferred from neighbouring sites
*wR*(*F*^2^) = 0.153	H atoms treated by a mixture of independent and constrained refinement
*S* = 1.05	*w* = 1/[σ^2^(*F*_o_^2^) + (0.085*P*)^2^ + 1.6883*P*] where *P* = (*F*_o_^2^ + 2*F*_c_^2^)/3
5066 reflections	(Δ/σ)_max_ < 0.001
288 parameters	Δρ_max_ = 0.32 e Å^−^^3^
0 restraints	Δρ_min_ = −0.27 e Å^−^^3^
Primary atom site location: structure-invariant direct methods	

### Special details

**Table:**

Geometry. All e.s.d.'s (except the e.s.d. in the dihedral angle between two l.s. planes) are estimated using the full covariance matrix. The cell e.s.d.'s are taken into account individually in the estimation of e.s.d.'s in distances, angles and torsion angles; correlations between e.s.d.'s in cell parameters are only used when they are defined by crystal symmetry. An approximate (isotropic) treatment of cell e.s.d.'s is used for estimating e.s.d.'s involving l.s. planes.
Refinement. Refinement was performed using all reflections. The weighted *R*-factor (*wR*) and goodness of fit (*S*) are based on *F*^2^. *R*-factor (gt) are based on *F*. The threshold expression of *F*^2^ > 2.0 σ(*F*^2^) is used only for calculating *R*-factor (gt).

### Fractional atomic coordinates and isotropic or equivalent isotropic displacement parameters (Å^2^)

**Table:**

	*x*	*y*	*z*	*U*_iso_*/*U*_eq_	
O1	0.17679 (3)	0.29705 (11)	0.33160 (7)	0.0425 (3)	
O2	0.10180 (4)	0.41560 (15)	0.56326 (9)	0.0605 (4)	
O3	0.20630 (5)	−0.00886 (16)	0.59592 (9)	0.0732 (5)	
O4	0.10890 (7)	−0.1543 (3)	0.70033 (15)	0.1349 (10)	
O5	0.05219 (8)	−0.1355 (3)	0.74228 (13)	0.1202 (8)	
N1	0.07269 (6)	−0.14573 (18)	0.68205 (12)	0.0698 (5)	
C1	0.14994 (4)	0.19968 (16)	0.49809 (10)	0.0386 (3)	
C2	0.11356 (4)	0.44403 (17)	0.49075 (10)	0.0434 (4)	
C3	0.10484 (5)	0.58616 (18)	0.44234 (12)	0.0513 (4)	
C4	0.10020 (5)	0.58137 (17)	0.33439 (11)	0.0447 (4)	
C5	0.13839 (5)	0.50909 (17)	0.31324 (11)	0.0457 (4)	
C6	0.23027 (5)	0.12986 (15)	0.34094 (11)	0.0426 (4)	
C7	0.24393 (5)	−0.01947 (16)	0.37621 (11)	0.0428 (4)	
C8	0.24960 (5)	−0.01955 (18)	0.48454 (11)	0.0489 (4)	
C9	0.21302 (5)	0.03331 (17)	0.52095 (11)	0.0453 (4)	
C10	0.13847 (4)	0.34177 (15)	0.45014 (10)	0.0376 (3)	
C11	0.15034 (4)	0.37823 (15)	0.37052 (10)	0.0378 (3)	
C12	0.19683 (4)	0.18691 (15)	0.38454 (10)	0.0372 (3)	
C13	0.18697 (4)	0.14200 (15)	0.46483 (10)	0.0374 (3)	
C14	0.11429 (5)	0.09666 (17)	0.47453 (11)	0.0415 (4)	
C15	0.09654 (5)	0.03555 (16)	0.53714 (10)	0.0410 (4)	
C16	0.06166 (4)	−0.06426 (15)	0.51520 (10)	0.0389 (3)	
C17	0.03628 (5)	−0.07231 (17)	0.42507 (11)	0.0458 (4)	
C18	0.00380 (5)	−0.1662 (2)	0.40412 (13)	0.0561 (5)	
C19	−0.00562 (6)	−0.2543 (2)	0.47284 (15)	0.0599 (5)	
C20	0.01737 (6)	−0.24595 (18)	0.56348 (14)	0.0565 (5)	
C21	0.05035 (5)	−0.15289 (16)	0.58291 (11)	0.0454 (4)	
C22	0.21140 (6)	−0.12792 (19)	0.33232 (14)	0.0595 (5)	
C23	0.28438 (6)	−0.0549 (2)	0.34786 (15)	0.0626 (5)	
C24	0.09700 (6)	0.73118 (19)	0.29204 (14)	0.0602 (5)	
C25	0.06161 (5)	0.4985 (3)	0.28947 (14)	0.0641 (5)	
H1	0.1573	0.2138	0.5668	0.0463*	
H3A	0.1270	0.6509	0.4687	0.0616*	
H3B	0.0798	0.6243	0.4564	0.0616*	
H5A	0.1332	0.4846	0.2466	0.0548*	
H5B	0.1611	0.5756	0.3258	0.0548*	
H6A	0.2537	0.1934	0.3554	0.0511*	
H6B	0.2207	0.1275	0.2727	0.0511*	
H8A	0.2558	−0.1157	0.5073	0.0587*	
H8B	0.2732	0.0392	0.5113	0.0587*	
H17	0.0414	−0.0126	0.3777	0.0549*	
H18	−0.0120	−0.1701	0.3428	0.0673*	
H19	−0.0273	−0.3185	0.4578	0.0718*	
H20	0.0109	−0.3019	0.6113	0.0678*	
H22A	0.1875	−0.1162	0.3587	0.0714*	
H22B	0.2222	−0.2221	0.3456	0.0714*	
H22C	0.2040	−0.1138	0.2651	0.0714*	
H23A	0.2918	−0.1515	0.3646	0.0751*	
H23B	0.3056	0.0074	0.3804	0.0751*	
H23C	0.2812	−0.0428	0.2807	0.0751*	
H24A	0.1214	0.7839	0.3190	0.0723*	
H24B	0.0735	0.7785	0.3059	0.0723*	
H24C	0.0942	0.7250	0.2247	0.0723*	
H25A	0.0379	0.5449	0.3035	0.0769*	
H25B	0.0635	0.4038	0.3145	0.0769*	
H25C	0.0591	0.4948	0.2221	0.0769*	
H14	0.1051 (7)	0.077 (3)	0.4067 (15)	0.067 (6)*	
H15	0.1074 (6)	0.060 (2)	0.6052 (14)	0.060 (6)*	

### Atomic displacement parameters (Å^2^)

**Table:**

	*U*^11^	*U*^22^	*U*^33^	*U*^12^	*U*^13^	*U*^23^
O1	0.0439 (6)	0.0459 (6)	0.0424 (6)	0.0097 (5)	0.0198 (5)	0.0088 (5)
O2	0.0655 (8)	0.0719 (8)	0.0529 (7)	0.0029 (7)	0.0325 (6)	−0.0044 (6)
O3	0.0850 (10)	0.0879 (10)	0.0501 (7)	0.0225 (8)	0.0219 (7)	0.0266 (7)
O4	0.0867 (14)	0.203 (3)	0.0983 (14)	−0.0202 (15)	−0.0178 (11)	0.0736 (16)
O5	0.170 (2)	0.1387 (19)	0.0595 (10)	−0.0029 (16)	0.0417 (12)	−0.0070 (11)
N1	0.0843 (13)	0.0648 (10)	0.0570 (10)	−0.0125 (9)	0.0078 (9)	0.0220 (8)
C1	0.0382 (8)	0.0456 (8)	0.0339 (7)	−0.0040 (6)	0.0123 (6)	0.0003 (6)
C2	0.0364 (8)	0.0538 (9)	0.0417 (8)	−0.0022 (7)	0.0121 (6)	−0.0079 (7)
C3	0.0501 (9)	0.0510 (9)	0.0545 (9)	0.0070 (7)	0.0149 (7)	−0.0080 (8)
C4	0.0376 (8)	0.0464 (8)	0.0502 (9)	0.0059 (6)	0.0095 (7)	−0.0004 (7)
C5	0.0421 (8)	0.0472 (9)	0.0507 (9)	0.0062 (7)	0.0163 (7)	0.0078 (7)
C6	0.0411 (8)	0.0415 (8)	0.0495 (9)	0.0003 (6)	0.0194 (7)	−0.0004 (7)
C7	0.0412 (8)	0.0401 (8)	0.0472 (8)	0.0013 (6)	0.0095 (6)	−0.0038 (7)
C8	0.0456 (9)	0.0484 (9)	0.0496 (9)	0.0083 (7)	0.0032 (7)	0.0012 (7)
C9	0.0484 (9)	0.0474 (9)	0.0384 (8)	−0.0001 (7)	0.0057 (7)	0.0020 (7)
C10	0.0319 (7)	0.0438 (8)	0.0384 (7)	−0.0020 (6)	0.0104 (6)	−0.0014 (6)
C11	0.0315 (7)	0.0424 (8)	0.0410 (7)	0.0005 (6)	0.0113 (6)	−0.0001 (6)
C12	0.0340 (7)	0.0380 (7)	0.0404 (7)	−0.0002 (6)	0.0100 (6)	0.0010 (6)
C13	0.0356 (7)	0.0401 (7)	0.0373 (7)	−0.0025 (6)	0.0096 (6)	−0.0012 (6)
C14	0.0407 (8)	0.0486 (8)	0.0365 (7)	−0.0052 (6)	0.0112 (6)	−0.0005 (7)
C15	0.0428 (8)	0.0438 (8)	0.0378 (8)	−0.0026 (6)	0.0120 (6)	0.0013 (7)
C16	0.0379 (7)	0.0385 (7)	0.0436 (8)	0.0024 (6)	0.0163 (6)	0.0002 (6)
C17	0.0449 (8)	0.0509 (9)	0.0438 (8)	0.0005 (7)	0.0146 (7)	0.0001 (7)
C18	0.0441 (9)	0.0627 (11)	0.0600 (10)	−0.0021 (8)	0.0083 (8)	−0.0075 (9)
C19	0.0433 (9)	0.0537 (10)	0.0849 (14)	−0.0093 (8)	0.0191 (9)	−0.0069 (10)
C20	0.0571 (11)	0.0459 (9)	0.0727 (12)	−0.0059 (8)	0.0279 (9)	0.0075 (9)
C21	0.0481 (9)	0.0421 (8)	0.0487 (9)	0.0012 (7)	0.0163 (7)	0.0052 (7)
C22	0.0687 (12)	0.0489 (9)	0.0596 (11)	−0.0118 (8)	0.0108 (9)	−0.0091 (8)
C23	0.0537 (11)	0.0621 (11)	0.0747 (12)	0.0149 (9)	0.0201 (9)	−0.0056 (10)
C24	0.0599 (11)	0.0530 (10)	0.0688 (12)	0.0158 (8)	0.0160 (9)	0.0065 (9)
C25	0.0437 (10)	0.0749 (13)	0.0682 (12)	0.0002 (9)	0.0001 (8)	−0.0048 (10)

### Geometric parameters (Å, º)

**Table:**

O1—C11	1.3747 (19)	C18—C19	1.381 (3)
O1—C12	1.3738 (17)	C19—C20	1.371 (3)
O2—C2	1.225 (2)	C20—C21	1.386 (3)
O3—C9	1.219 (3)	C1—H1	0.980
O4—N1	1.181 (3)	C3—H3A	0.970
O5—N1	1.220 (4)	C3—H3B	0.970
N1—C21	1.468 (3)	C5—H5A	0.970
C1—C10	1.517 (2)	C5—H5B	0.970
C1—C13	1.517 (2)	C6—H6A	0.970
C1—C14	1.514 (3)	C6—H6B	0.970
C2—C3	1.509 (3)	C8—H8A	0.970
C2—C10	1.473 (3)	C8—H8B	0.970
C3—C4	1.536 (3)	C14—H14	0.98 (2)
C4—C5	1.530 (3)	C15—H15	1.001 (19)
C4—C24	1.532 (3)	C17—H17	0.930
C4—C25	1.525 (3)	C18—H18	0.930
C5—C11	1.490 (2)	C19—H19	0.930
C6—C7	1.531 (2)	C20—H20	0.930
C6—C12	1.493 (3)	C22—H22A	0.960
C7—C8	1.537 (3)	C22—H22B	0.960
C7—C22	1.525 (3)	C22—H22C	0.960
C7—C23	1.527 (3)	C23—H23A	0.960
C8—C9	1.512 (3)	C23—H23B	0.960
C9—C13	1.468 (2)	C23—H23C	0.960
C10—C11	1.340 (3)	C24—H24A	0.960
C12—C13	1.341 (3)	C24—H24B	0.960
C14—C15	1.315 (3)	C24—H24C	0.960
C15—C16	1.475 (2)	C25—H25A	0.960
C16—C17	1.395 (2)	C25—H25B	0.960
C16—C21	1.398 (3)	C25—H25C	0.960
C17—C18	1.379 (3)		
			
O1···C1	2.8912 (19)	O3···H6B^iv^	2.7353
O2···C1	2.871 (2)	O3···H22B^xi^	3.4562
O2···C11	3.527 (3)	O3···H22C^iv^	2.7199
O2···C14	3.326 (3)	O3···H23A^xi^	3.2460
O2···C15	3.598 (2)	O3···H23C^iv^	3.2773
O3···C1	2.871 (2)	O4···H5A^iv^	3.2458
O3···C12	3.525 (2)	O4···H25B^iv^	3.4054
O3···C14	3.340 (3)	O4···H14^iv^	3.10 (3)
O3···C15	3.596 (3)	O5···H17^iii^	3.5151
O4···C15	2.918 (3)	O5···H17^iv^	2.4916
O4···C16	2.924 (3)	O5···H18^iii^	3.2981
O4···C20	3.371 (3)	O5···H24B^x^	3.5167
O5···C16	3.431 (3)	O5···H25B^iv^	2.7278
O5···C20	2.797 (3)	O5···H25C^iv^	3.4071
N1···C15	2.941 (3)	O5···H14^iv^	2.69 (2)
C2···C5	2.925 (3)	N1···H17^iv^	3.5458
C2···C14	3.279 (3)	N1···H18^iii^	3.5702
C2···C25	3.093 (3)	N1···H25B^iv^	3.1486
C3···C11	2.808 (3)	N1···H25C^iv^	3.3845
C4···C10	2.932 (2)	N1···H14^iv^	3.26 (2)
C6···C9	2.928 (3)	C1···H6A^ix^	3.5897
C7···C13	2.932 (3)	C1···H8B^ix^	3.5738
C8···C12	2.805 (2)	C1···H23B^ix^	3.4306
C9···C14	3.267 (3)	C2···H19^iii^	3.3364
C9···C22	3.112 (3)	C2···H23B^ix^	2.9634
C10···C12	2.756 (2)	C5···H8B^ix^	3.4808
C10···C15	3.549 (3)	C5···H23C^ii^	3.2897
C10···C25	3.402 (3)	C6···H23A^ii^	3.5589
C11···C13	2.753 (2)	C6···H23C^ii^	3.5284
C11···C14	3.392 (3)	C8···H8A^xi^	3.4423
C11···C25	3.147 (2)	C8···H22B^xi^	3.4415
C12···C14	3.393 (3)	C9···H6A^ix^	3.1931
C12···C22	3.122 (3)	C10···H8B^ix^	3.0866
C13···C15	3.539 (3)	C10···H23B^ix^	3.0874
C13···C22	3.385 (3)	C11···H8B^ix^	2.8547
C14···C17	3.000 (3)	C11···H23C^ii^	3.5605
C16···C19	2.830 (3)	C12···H8B^ix^	3.0450
C17···C20	2.759 (3)	C13···H6A^ix^	3.2954
C18···C21	2.717 (3)	C13···H8B^ix^	3.2704
O1···O4^i^	2.946 (3)	C15···H19^iii^	3.5356
O1···C23^ii^	3.434 (3)	C15···H24C^x^	3.5397
O2···C19^iii^	3.482 (3)	C16···H3B^vi^	3.1469
O4···O1^iv^	2.946 (3)	C16···H24B^vi^	3.4656
O4···C11^iv^	3.308 (3)	C17···H3B^vi^	3.1904
O4···C12^iv^	3.524 (3)	C17···H24B^vi^	2.7183
O5···O5^v^	3.532 (4)	C18···H3B^vi^	3.1692
O5···C14^iv^	3.565 (3)	C18···H24B^vi^	3.0142
O5···C17^iv^	3.420 (3)	C18···H24B^xii^	3.5727
O5···C25^iv^	3.486 (4)	C18···H24C^xii^	3.5436
C11···O4^i^	3.308 (3)	C18···H25A^vi^	3.3944
C12···O4^i^	3.524 (3)	C19···H3B^vi^	3.1212
C14···O5^i^	3.565 (3)	C20···H3B^vi^	3.0969
C16···C18^iii^	3.452 (3)	C20···H17^iii^	3.3465
C16···C19^iii^	3.557 (3)	C20···H25C^iv^	3.3632
C17···O5^i^	3.420 (3)	C21···H3B^vi^	3.0783
C17···C20^iii^	3.511 (3)	C21···H17^iii^	3.5840
C17···C21^iii^	3.563 (3)	C21···H18^iii^	3.5529
C17···C24^vi^	3.589 (3)	C22···H5B^vi^	3.2455
C18···C16^iii^	3.452 (3)	C22···H6A^vii^	3.5913
C18···C21^iii^	3.529 (3)	C22···H8A^xi^	3.3626
C19···O2^iii^	3.482 (3)	C22···H24A^vi^	3.0775
C19···C16^iii^	3.557 (3)	C23···H5A^vii^	3.3396
C20···C17^iii^	3.511 (3)	C23···H6B^vii^	3.4470
C21···C17^iii^	3.563 (3)	C24···H17^viii^	3.4298
C21···C18^iii^	3.529 (3)	C24···H18^xiii^	3.2108
C23···O1^vii^	3.434 (3)	C24···H22A^viii^	3.2921
C24···C17^viii^	3.589 (3)	C24···H15^xiv^	3.42 (2)
C25···O5^i^	3.486 (4)	C25···H20^iii^	3.5796
O1···H5A	2.4439	C25···H20^i^	3.3237
O1···H5B	2.6718	C25···H25A^xv^	3.3288
O1···H6A	2.6941	H1···H6A^ix^	3.0646
O1···H6B	2.4398	H1···H22C^iv^	3.1000
O1···H14	3.50 (3)	H1···H23B^ix^	2.9325
O2···H1	2.6432	H1···H24C^x^	3.4669
O2···H3A	2.8236	H3A···H22A^viii^	3.5743
O2···H3B	2.5103	H3A···H23A^ix^	3.2141
O2···H25B	3.5502	H3A···H23B^ix^	3.1495
O2···H15	3.40 (2)	H3B···C16^viii^	3.1469
O3···H1	2.6349	H3B···C17^viii^	3.1904
O3···H8A	2.5055	H3B···C18^viii^	3.1692
O3···H8B	2.7992	H3B···C19^viii^	3.1212
O3···H22A	3.5002	H3B···C20^viii^	3.0969
O3···H15	3.39 (2)	H3B···C21^viii^	3.0783
O4···H20	3.5265	H3B···H20^viii^	3.5998
O4···H15	2.44 (2)	H5A···O2^xiv^	2.7946
O5···H20	2.6105	H5A···O4^i^	3.2458
O5···H15	3.50 (3)	H5A···C23^ii^	3.3396
N1···H20	2.5572	H5A···H23A^ii^	3.4923
N1···H15	2.63 (2)	H5A···H23B^ii^	3.0312
C1···H15	2.66 (3)	H5A···H23C^ii^	2.9733
C2···H1	2.7095	H5B···C22^viii^	3.2455
C2···H5B	3.3702	H5B···H8A^ix^	3.2789
C2···H25A	3.4131	H5B···H8B^ix^	3.0487
C2···H25B	2.7554	H5B···H22A^viii^	3.0393
C3···H5A	3.3114	H5B···H22B^viii^	2.7555
C3···H5B	2.7791	H5B···H22C^viii^	3.4496
C3···H24A	2.7150	H5B···H23B^ii^	3.4571
C3···H24B	2.7158	H5B···H23C^ii^	2.9283
C3···H24C	3.3556	H6A···O3^ix^	3.2694
C3···H25A	2.6884	H6A···C1^ix^	3.5897
C3···H25B	2.6773	H6A···C9^ix^	3.1931
C3···H25C	3.3346	H6A···C13^ix^	3.2954
C5···H3A	2.7099	H6A···C22^ii^	3.5913
C5···H3B	3.3179	H6A···H1^ix^	3.0646
C5···H24A	2.6534	H6A···H8B^ix^	3.4060
C5···H24B	3.3203	H6A···H22B^ii^	3.2741
C5···H24C	2.6711	H6A···H22C^ii^	3.0596
C5···H25A	3.3359	H6A···H23A^ii^	3.5312
C5···H25B	2.6881	H6A···H23C^ii^	3.2265
C5···H25C	2.6911	H6B···O3^i^	2.7353
C6···H8A	3.3131	H6B···C23^ii^	3.4470
C6···H8B	2.7064	H6B···H22B^ii^	3.1533
C6···H22A	2.7600	H6B···H23A^ii^	2.8434
C6···H22B	3.3260	H6B···H23C^ii^	3.1963
C6···H22C	2.6111	H8A···C8^xi^	3.4423
C6···H23A	3.3216	H8A···C22^xi^	3.3626
C6···H23B	2.7079	H8A···H5B^ix^	3.2789
C6···H23C	2.6291	H8A···H8A^xi^	2.5608
C8···H6A	2.7633	H8A···H22A^xi^	3.4811
C8···H6B	3.3104	H8A···H22B^xi^	2.5932
C8···H22A	2.6076	H8A···H23A^xi^	3.4651
C8···H22B	2.7784	H8B···O1^ix^	2.9483
C8···H22C	3.3309	H8B···C1^ix^	3.5738
C8···H23A	2.7521	H8B···C5^ix^	3.4808
C8···H23B	2.6478	H8B···C10^ix^	3.0866
C8···H23C	3.3421	H8B···C11^ix^	2.8547
C9···H1	2.7026	H8B···C12^ix^	3.0450
C9···H6A	3.3516	H8B···C13^ix^	3.2704
C9···H22A	2.7108	H8B···H5B^ix^	3.0487
C9···H22B	3.5546	H8B···H6A^ix^	3.4060
C10···H3A	2.9546	H17···O5^iii^	3.5151
C10···H3B	3.3143	H17···O5^i^	2.4916
C10···H5A	3.2062	H17···N1^i^	3.5458
C10···H5B	3.0385	H17···C20^iii^	3.3465
C10···H25B	2.8843	H17···C21^iii^	3.5840
C10···H14	2.74 (2)	H17···C24^vi^	3.4298
C11···H1	3.1975	H17···H18^xv^	3.4610
C11···H3A	3.1115	H17···H20^iii^	3.4577
C11···H25B	2.8416	H17···H24A^vi^	3.5294
C11···H25C	3.5005	H17···H24B^vi^	2.5609
C11···H14	3.30 (2)	H18···O5^iii^	3.2981
C12···H1	3.1995	H18···N1^iii^	3.5702
C12···H8B	3.1239	H18···C21^iii^	3.5529
C12···H22A	2.8858	H18···C24^xii^	3.2108
C12···H22C	3.3516	H18···H17^xv^	3.4610
C12···H14	3.30 (3)	H18···H18^xv^	2.9593
C13···H6A	3.0319	H18···H24B^vi^	3.0455
C13···H6B	3.2160	H18···H24B^xii^	2.6754
C13···H8A	3.3039	H18···H24C^xii^	2.8754
C13···H8B	2.9675	H18···H25A^vi^	3.2705
C13···H22A	2.8763	H18···H25A^xii^	3.4107
C13···H14	2.74 (2)	H18···H25C^xii^	3.5574
C14···H17	2.7299	H18···H15^iii^	3.5711
C15···H1	2.5922	H19···O2^iii^	2.6018
C15···H17	2.6598	H19···C2^iii^	3.3364
C16···H18	3.2566	H19···C15^iii^	3.5356
C16···H20	3.2862	H19···H24C^xii^	3.0939
C16···H14	2.70 (3)	H19···H25C^xii^	3.1296
C17···H19	3.2415	H19···H15^iii^	3.5795
C17···H14	2.75 (3)	H20···C25^iii^	3.5796
C17···H15	3.363 (18)	H20···C25^iv^	3.3237
C18···H20	3.2178	H20···H3B^vi^	3.5998
C19···H17	3.2330	H20···H17^iii^	3.4577
C20···H18	3.2108	H20···H25A^iii^	3.2033
C21···H17	3.2027	H20···H25A^iv^	3.5624
C21···H19	3.2217	H20···H25B^iii^	3.0597
C21···H15	2.74 (2)	H20···H25B^iv^	3.2270
C22···H6A	3.3236	H20···H25C^iv^	2.7081
C22···H6B	2.5953	H22A···C24^vi^	3.2921
C22···H8A	2.6456	H22A···H3A^vi^	3.5743
C22···H8B	3.3342	H22A···H5B^vi^	3.0393
C22···H23A	2.6269	H22A···H8A^xi^	3.4811
C22···H23B	3.3183	H22A···H24A^vi^	2.3506
C22···H23C	2.7109	H22B···O3^xi^	3.4562
C23···H6A	2.5625	H22B···C8^xi^	3.4415
C23···H6B	2.7632	H22B···H5B^vi^	2.7555
C23···H8A	2.7372	H22B···H6A^vii^	3.2741
C23···H8B	2.6236	H22B···H6B^vii^	3.1533
C23···H22A	3.3149	H22B···H8A^xi^	2.5932
C23···H22B	2.5968	H22B···H23C^vii^	3.5180
C23···H22C	2.7443	H22B···H24A^vi^	3.2970
C24···H3A	2.6457	H22C···O3^i^	2.7199
C24···H3B	2.7518	H22C···H1^i^	3.1000
C24···H5A	2.7594	H22C···H5B^vi^	3.4496
C24···H5B	2.5496	H22C···H6A^vii^	3.0596
C24···H25A	2.6674	H22C···H24A^vi^	3.1680
C24···H25B	3.3178	H22C···H15^i^	3.5723
C24···H25C	2.6499	H23A···O1^vii^	3.2632
C25···H3A	3.3310	H23A···O3^xi^	3.2460
C25···H3B	2.6391	H23A···C6^vii^	3.5589
C25···H5A	2.5925	H23A···H3A^ix^	3.2141
C25···H5B	3.3239	H23A···H5A^vii^	3.4923
C25···H24A	3.3164	H23A···H6A^vii^	3.5312
C25···H24B	2.6681	H23A···H6B^vii^	2.8434
C25···H24C	2.6528	H23A···H8A^xi^	3.4651
H1···H14	2.8809	H23B···O2^ix^	3.1035
H1···H15	2.3552	H23B···C1^ix^	3.4306
H3A···H5B	2.6594	H23B···C2^ix^	2.9634
H3A···H24A	2.4729	H23B···C10^ix^	3.0874
H3A···H24B	2.8917	H23B···H1^ix^	2.9325
H3A···H24C	3.5323	H23B···H3A^ix^	3.1495
H3A···H25A	3.5304	H23B···H5A^vii^	3.0312
H3A···H25B	3.5823	H23B···H5B^vii^	3.4571
H3B···H24A	3.0507	H23C···O1^vii^	2.8010
H3B···H24B	2.5856	H23C···O3^i^	3.2773
H3B···H25A	2.4626	H23C···C5^vii^	3.2897
H3B···H25B	2.8870	H23C···C6^vii^	3.5284
H3B···H25C	3.5259	H23C···C11^vii^	3.5605
H5A···H24A	3.0605	H23C···H5A^vii^	2.9733
H5A···H24C	2.5958	H23C···H5B^vii^	2.9283
H5A···H25A	3.4909	H23C···H6A^vii^	3.2265
H5A···H25B	2.8125	H23C···H6B^vii^	3.1963
H5A···H25C	2.4196	H23C···H22B^ii^	3.5180
H5B···H24A	2.3562	H24A···C22^viii^	3.0775
H5B···H24B	3.4498	H24A···H17^viii^	3.5294
H5B···H24C	2.7739	H24A···H22A^viii^	2.3506
H5B···H25C	3.4928	H24A···H22B^viii^	3.2970
H6A···H8B	2.6423	H24A···H22C^viii^	3.1680
H6A···H22C	3.4467	H24A···H14^viii^	3.1352
H6A···H23A	3.4783	H24A···H15^xiv^	3.3614
H6A···H23B	2.4317	H24B···O5^xiv^	3.5167
H6A···H23C	2.7150	H24B···C16^viii^	3.4656
H6B···H22A	2.9368	H24B···C17^viii^	2.7183
H6B···H22B	3.4524	H24B···C18^viii^	3.0142
H6B···H22C	2.3353	H24B···C18^xiii^	3.5727
H6B···H23A	3.5939	H24B···H17^viii^	2.5609
H6B···H23B	3.1308	H24B···H18^viii^	3.0455
H6B···H23C	2.5573	H24B···H18^xiii^	2.6754
H8A···H22A	2.7741	H24B···H14^viii^	3.2391
H8A···H22B	2.5702	H24C···O2^xiv^	2.7421
H8A···H22C	3.5574	H24C···C15^xiv^	3.5397
H8A···H23A	2.6218	H24C···C18^xiii^	3.5436
H8A···H23B	2.9602	H24C···H1^xiv^	3.4669
H8B···H22A	3.5276	H24C···H18^xiii^	2.8754
H8B···H22B	3.5955	H24C···H19^xiii^	3.0939
H8B···H23A	2.9446	H24C···H15^xiv^	2.7534
H8B···H23B	2.3893	H25A···C18^viii^	3.3944
H8B···H23C	3.4868	H25A···C25^xv^	3.3288
H17···H18	2.2884	H25A···H18^viii^	3.2705
H17···H14	2.2395	H25A···H18^xiii^	3.4107
H18···H19	2.3106	H25A···H20^iii^	3.2033
H19···H20	2.3136	H25A···H20^i^	3.5624
H22A···H23A	3.4724	H25A···H25A^xv^	2.6653
H22A···H14	3.4855	H25A···H25C^xv^	3.2077
H22B···H23A	2.3707	H25B···O4^i^	3.4054
H22B···H23B	3.4697	H25B···O5^i^	2.7278
H22B···H23C	2.8956	H25B···N1^i^	3.1486
H22C···H23A	2.9896	H25B···H20^iii^	3.0597
H22C···H23C	2.6188	H25B···H20^i^	3.2270
H24A···H25A	3.5458	H25C···O2^xiv^	3.0683
H24A···H25C	3.5254	H25C···O5^i^	3.4071
H24B···H25A	2.4942	H25C···N1^i^	3.3845
H24B···H25B	3.5482	H25C···C20^i^	3.3632
H24B···H25C	2.9295	H25C···H18^xiii^	3.5574
H24C···H25A	2.9326	H25C···H19^xiii^	3.1296
H24C···H25B	3.5283	H25C···H20^i^	2.7081
H24C···H25C	2.4591	H25C···H25A^xv^	3.2077
H25B···H14	3.5141	H14···O4^i^	3.10 (3)
H14···H15	2.86 (3)	H14···O5^i^	2.69 (2)
O1···H8B^ix^	2.9483	H14···N1^i^	3.26 (2)
O1···H23A^ii^	3.2632	H14···H24A^vi^	3.1352
O1···H23C^ii^	2.8010	H14···H24B^vi^	3.2391
O2···H5A^x^	2.7946	H15···C24^x^	3.42 (2)
O2···H19^iii^	2.6018	H15···H18^iii^	3.5711
O2···H23B^ix^	3.1035	H15···H19^iii^	3.5795
O2···H24C^x^	2.7421	H15···H22C^iv^	3.5723
O2···H25C^x^	3.0683	H15···H24A^x^	3.3614
O3···H6A^ix^	3.2694	H15···H24C^x^	2.7534
			
C11—O1—C12	117.97 (12)	C2—C3—H3A	108.664
O4—N1—O5	123.2 (2)	C2—C3—H3B	108.668
O4—N1—C21	119.7 (2)	C4—C3—H3A	108.660
O5—N1—C21	117.15 (19)	C4—C3—H3B	108.666
C10—C1—C13	108.23 (13)	H3A—C3—H3B	107.605
C10—C1—C14	110.70 (11)	C4—C5—H5A	108.979
C13—C1—C14	110.56 (13)	C4—C5—H5B	108.973
O2—C2—C3	121.71 (15)	C11—C5—H5A	108.980
O2—C2—C10	120.62 (15)	C11—C5—H5B	108.977
C3—C2—C10	117.61 (14)	H5A—C5—H5B	107.787
C2—C3—C4	114.37 (14)	C7—C6—H6A	109.061
C3—C4—C5	108.03 (13)	C7—C6—H6B	109.060
C3—C4—C24	111.24 (14)	C12—C6—H6A	109.064
C3—C4—C25	109.86 (15)	C12—C6—H6B	109.075
C5—C4—C24	108.60 (15)	H6A—C6—H6B	107.811
C5—C4—C25	110.40 (14)	C7—C8—H8A	108.514
C24—C4—C25	108.70 (14)	C7—C8—H8B	108.512
C4—C5—C11	113.01 (14)	C9—C8—H8A	108.525
C7—C6—C12	112.65 (14)	C9—C8—H8B	108.528
C6—C7—C8	107.62 (13)	H8A—C8—H8B	107.507
C6—C7—C22	110.02 (13)	C1—C14—H14	113.9 (13)
C6—C7—C23	109.11 (15)	C15—C14—H14	121.2 (13)
C8—C7—C22	110.17 (15)	C14—C15—H15	117.0 (12)
C8—C7—C23	110.54 (14)	C16—C15—H15	117.6 (12)
C22—C7—C23	109.35 (15)	C16—C17—H17	119.000
C7—C8—C9	115.01 (13)	C18—C17—H17	119.023
O3—C9—C8	121.19 (15)	C17—C18—H18	119.485
O3—C9—C13	121.24 (17)	C19—C18—H18	119.482
C8—C9—C13	117.51 (14)	C18—C19—H19	120.454
C1—C10—C2	120.15 (14)	C20—C19—H19	120.441
C1—C10—C11	121.78 (14)	C19—C20—H20	120.458
C2—C10—C11	118.07 (13)	C21—C20—H20	120.461
O1—C11—C5	110.51 (13)	C7—C22—H22A	109.462
O1—C11—C10	122.86 (13)	C7—C22—H22B	109.482
C5—C11—C10	126.62 (14)	C7—C22—H22C	109.478
O1—C12—C6	110.72 (13)	H22A—C22—H22B	109.463
O1—C12—C13	122.70 (14)	H22A—C22—H22C	109.456
C6—C12—C13	126.57 (13)	H22B—C22—H22C	109.486
C1—C13—C9	119.90 (14)	C7—C23—H23A	109.465
C1—C13—C12	121.90 (13)	C7—C23—H23B	109.469
C9—C13—C12	118.20 (14)	C7—C23—H23C	109.458
C1—C14—C15	124.89 (14)	H23A—C23—H23B	109.488
C14—C15—C16	125.48 (14)	H23A—C23—H23C	109.468
C15—C16—C17	121.81 (14)	H23B—C23—H23C	109.479
C15—C16—C21	123.22 (13)	C4—C24—H24A	109.472
C17—C16—C21	114.92 (13)	C4—C24—H24B	109.473
C16—C17—C18	121.98 (16)	C4—C24—H24C	109.477
C17—C18—C19	121.03 (16)	H24A—C24—H24B	109.466
C18—C19—C20	119.10 (18)	H24A—C24—H24C	109.470
C19—C20—C21	119.08 (18)	H24B—C24—H24C	109.470
N1—C21—C16	120.00 (14)	C4—C25—H25A	109.470
N1—C21—C20	116.14 (16)	C4—C25—H25B	109.480
C16—C21—C20	123.80 (15)	C4—C25—H25C	109.479
C10—C1—H1	109.114	H25A—C25—H25B	109.468
C13—C1—H1	109.098	H25A—C25—H25C	109.463
C14—C1—H1	109.114	H25B—C25—H25C	109.467
			
C11—O1—C12—C6	−168.31 (10)	C12—C6—C7—C8	−46.35 (15)
C11—O1—C12—C13	10.75 (17)	C12—C6—C7—C22	73.70 (15)
C12—O1—C11—C5	168.03 (10)	C12—C6—C7—C23	−166.33 (10)
C12—O1—C11—C10	−10.74 (17)	C6—C7—C8—C9	53.11 (17)
O4—N1—C21—C16	−51.8 (3)	C22—C7—C8—C9	−66.85 (17)
O4—N1—C21—C20	130.9 (3)	C23—C7—C8—C9	172.18 (13)
O5—N1—C21—C16	130.5 (2)	C7—C8—C9—O3	151.50 (14)
O5—N1—C21—C20	−46.8 (3)	C7—C8—C9—C13	−31.18 (19)
C10—C1—C13—C9	159.62 (10)	O3—C9—C13—C1	−2.4 (2)
C10—C1—C13—C12	−20.69 (16)	O3—C9—C13—C12	177.92 (14)
C13—C1—C10—C2	−159.45 (10)	C8—C9—C13—C1	−179.69 (11)
C13—C1—C10—C11	20.69 (16)	C8—C9—C13—C12	0.61 (19)
C10—C1—C14—C15	−120.69 (15)	C1—C10—C11—O1	−6.46 (19)
C14—C1—C10—C2	79.24 (15)	C1—C10—C11—C5	174.98 (11)
C14—C1—C10—C11	−100.62 (15)	C2—C10—C11—O1	173.67 (11)
C13—C1—C14—C15	119.39 (16)	C2—C10—C11—C5	−4.89 (19)
C14—C1—C13—C9	−78.98 (14)	O1—C12—C13—C1	6.4 (2)
C14—C1—C13—C12	100.70 (15)	O1—C12—C13—C9	−173.88 (10)
O2—C2—C3—C4	−149.19 (13)	C6—C12—C13—C1	−174.67 (11)
O2—C2—C10—C1	−0.14 (19)	C6—C12—C13—C9	5.0 (2)
O2—C2—C10—C11	179.73 (12)	C1—C14—C15—C16	179.61 (13)
C3—C2—C10—C1	177.22 (11)	C14—C15—C16—C17	−19.3 (3)
C3—C2—C10—C11	−2.92 (17)	C14—C15—C16—C21	163.59 (15)
C10—C2—C3—C4	33.48 (17)	C15—C16—C17—C18	179.56 (13)
C2—C3—C4—C5	−53.53 (17)	C15—C16—C21—N1	2.2 (3)
C2—C3—C4—C24	−172.64 (12)	C15—C16—C21—C20	179.23 (13)
C2—C3—C4—C25	66.95 (16)	C17—C16—C21—N1	−175.15 (13)
C3—C4—C5—C11	45.07 (16)	C17—C16—C21—C20	1.9 (3)
C24—C4—C5—C11	165.84 (12)	C21—C16—C17—C18	−3.1 (3)
C25—C4—C5—C11	−75.08 (16)	C16—C17—C18—C19	1.7 (3)
C4—C5—C11—O1	163.21 (11)	C17—C18—C19—C20	1.2 (3)
C4—C5—C11—C10	−18.08 (19)	C18—C19—C20—C21	−2.3 (3)
C7—C6—C12—O1	−161.08 (11)	C19—C20—C21—N1	177.90 (16)
C7—C6—C12—C13	19.91 (19)	C19—C20—C21—C16	0.7 (3)

Symmetry codes: (i) *x*, −*y*, *z*−1/2; (ii) −*x*+1/2, *y*+1/2, −*z*+1/2; (iii) −*x*, −*y*, −*z*+1; (iv) *x*, −*y*, *z*+1/2; (v) −*x*, *y*, −*z*+3/2; (vi) *x*, *y*−1, *z*; (vii) −*x*+1/2, *y*−1/2, −*z*+1/2; (viii) *x*, *y*+1, *z*; (ix) −*x*+1/2, −*y*+1/2, −*z*+1; (x) *x*, −*y*+1, *z*+1/2; (xi) −*x*+1/2, −*y*−1/2, −*z*+1; (xii) −*x*, *y*−1, −*z*+1/2; (xiii) −*x*, *y*+1, −*z*+1/2; (xiv) *x*, −*y*+1, *z*−1/2; (xv) −*x*, *y*, −*z*+1/2.

### Hydrogen-bond geometry (Å, º)

**Table:**

*D*—H···*A*	*D*—H	H···*A*	*D*···*A*	*D*—H···*A*
C17—H17···O5^i^	0.93	2.49	3.420 (3)	176

Symmetry code: (i) *x*, −*y*, *z*−1/2.
